# Clinical presentation and outcomes of patients with biallelic *SCN5A* variants: A systematic review

**DOI:** 10.1016/j.hroo.2025.06.010

**Published:** 2025-06-20

**Authors:** Elodie Surget, Alexis Hermida, Alice Maltret, James Marchant, Isabelle Denjoy, Laure Champ-Rigot, Estelle Gandjbakhch, Charles Morgat, Alessandra Pia Porretta, Véronique Fressart, Antoine Leenhardt, Fabrice Extramiana

**Affiliations:** 1 Reference Center for Inherited Arrhythmic Syndromes, Hôpital Bichat, APHP, Université Paris Cité, Paris, France; 2 Centre hospitalier universitaire Robert Debré, Université Paris Cité, Paris, France; 3 Centre hospitalier universitaire Amiens-Picardie, CHU Amiens, Amiens, France; 4 Department of Congenital Heart Diseases, M3C-Marie Lannelongue, Hôpital Marie Lannelongue, Groupe Hospitalier Paris Saint Joseph, Le Plessis Robinson, France; 5 Development, Aging and Regeneration Program, Center for Genetic Disorders and Aging Research, Sanford Burnham Prebys Medical Discovery Institute, La Jolla, California; 6 Normandie Univ, UNICAEN, CHU de Caen Normandie, Service de Cardiologie, Caen, France; 7 Department of Cardiology, La Pitié-Salpêtrière Hospital, Paris, France; 8 Service of Cardiology, Heart and Vessel Department, Centre Hospitalier Universitaire Vaudois, Lausanne, Switzerland; 9 APHP, Referral Center for Cardiac Hereditary Diseases, Sorbonne University, Pitié-Salpêtrière Hospital, Paris, France

**Keywords:** *SCN5A*, Sudden death, Atrial standstill, Stroke, Brugada

## Abstract

**Background:**

Heterozygous *SCN5A* variants are associated with a wide spectrum of inherited arrhythmic disorders. However, little is known about the clinical phenotypes associated with biallelic (ie, homozygous or compound heterozygous) *SCN5A* variants.

**Objective:**

The purpose of this study was to systematically review the clinical characteristics, outcomes, and genotype-phenotype correlations in patients with biallelic *SCN5A* variants.

**Methods:**

We reviewed the available literature on patients with biallelic *SCN5A* variants and evaluated their demographic characteristics, medical history, and prognosis.

**Results:**

A total of 33 articles were selected, comprising 61 patients from 43 families. Most were symptomatic at diagnosis (29/36, 81%), with common presentations including syncope (15/36, 42%) and cardiac arrest (10/36, 28%). Sinus node dysfunction was the most prevalent phenotype (35/54, 65%), often associated with atrial standstill (20/34, 59%). Other electrocardiographic abnormalities included progressive cardiac conduction disease (17/52, 33%), Brugada syndrome (12/52, 23%), overlap phenotypes (7/52, 13%), and long QT syndrome (2/52, 4%). During follow-up (median 5 ± 7 years), arrhythmias were common, and 76% (39/51) of patients required device implantation. Major cardiac events occurred in 50%, and 15% (8/55) died at a young age (mean 9 ± 15 years). Genotype analysis revealed 21 patients with homozygous *SCN5A* variants (34%) and 40 with compound heterozygous variants (66%). The type of variant, rather than zygosity, correlated with prognosis: a non-missense variant was associated with earlier onset and increased risk of major cardiac events.

**Conclusion:**

Patients with biallelic *SCN5A* variants display early-onset severe arrhythmic phenotypes with high morbidity and mortality. Improved recognition and international collaboration are essential to optimize diagnosis, management, and prevention of sudden cardiac death in this rare but high-risk population.

**Key Findings**

▪ The most frequent cardiac phenotypes in patients with biallelic *SCN5A* variants are sinus node dysfunction, progressive cardiac conduction disease, and Brugada syndrome.
▪ Patients with biallelic *SCN5A* variants frequently present with early-onset and severe cardiac phenotypes.
▪ Non-missense variants are associated with worse outcomes. They are significantly associated with younger age at diagnosis, proband status, and a higher risk of major cardiac events.
▪ Despite a high prevalence of stroke, only 5% of patients received anticoagulation, suggesting that the risk of cardioembolic complications may be underestimated in this population.

## Introduction

The voltage-gated cardiac sodium channel Na_V_1.5 is involved in phase 0 of the action potential and is essential for the initiation and propagation of cardiac depolarization.^1^ Variants in the *SCN5A* gene, which encodes the cardiac sodium channel α subunit, are associated with a wide spectrum of inherited arrhythmic disorders. Gain-of-function variants that increase the late inward sodium current are associated with long QT syndrome (LQTS) type 3^2^ and multifocal ectopic premature Purkinje-related complexes.^3^ Conversely, loss-of-function variants, leading to a decreased peak late inward sodium current, are associated with multiple clinical phenotypes, such as Brugada syndrome (BrS),^4^ progressive cardiac conduction disease (PCCD),^5^ sinus node dysfunction (SND),^6^ atrial fibrillation,^7^ and dilated cardiomyopathy.^8^ However, a single *SCN5A* variant may confer heterogeneous biophysical properties, which, by accounting for both gain- and loss-of-function effects, may lead to overlapping phenotypes.^9^

Although several studies have already described the clinical characteristics of heterozygous *SCN5A* variants,^10^, ^11^, ^12^ little is known about biallelic *SCN5A* variants. Hence, we performed a literature review to evaluate the clinical presentation and prognosis of these patients.

## Methods

### Protocol and registration

This systematic review was designed in accordance with the Preferred Reporting Items for Systematic Reviews and Meta Analyses guidelines.^13^ The review protocol (registration number CRD420251012866) was registered in the International Prospective Register of Systematic Reviews.

### Eligibility criteria

Inclusion criteria were established as follows: articles published in English that included patients with pathogenic or likely pathogenic biallelic *SCN5A* variants. The pathogenicity of these variants was determined according to the American College of Medical Genetics and Genomics^14^ guidelines. Biallelic *SCN5A* variants included homozygous and compound heterozygous variants. Exclusion criteria encompassed articles involving patients with common genetic variants in *SCN5A* (eg, H558R or S1103Y) or variants in additional genes.

### Literature retrieval

A thorough literature search was performed using PubMed and Google Scholar from their inception to February 2025. The search strategy incorporated the MeSH (Medical Subject Headings) terms “homozygous *SCN5A* variant” or “compound heterozygote *SCN5A* variant.” Reference lists of the included articles were also scanned to identify any studies that may have been missed during the initial search.

### Study selection and data extraction

Duplicate records were identified and removed. Full-text articles of potentially relevant studies were assessed for inclusion. Data extraction included data on demographic and clinical characteristics (age, sex, symptoms, etc), electrocardiography (ECG), echocardiography, and treatment (antiarrhythmics, anticoagulation, and device implantation). The prognosis of patients with biallelic *SCN5A* variants was evaluated on the basis of the occurrence of major cardiac events (MCEs; including ventricular tachycardia, cardiac arrest [CA], or death) and stroke.

### Statistical analysis

Continuous variables are presented as mean ± standard deviation and categorical variables as count and percentage. Comparisons between categorical variables were performed using the χ^2^ test or Fisher exact test, as appropriate. For continuous variables, the Mann-Whitney *U* test was used for comparisons between 2 groups and the Kruskal-Wallis test for comparisons among more than 2 groups.

Logistic regression analyses were performed to assess associations between categorical variables and clinical outcomes. Binary logistic regression was used for variables with 2 categories, while multinomial logistic regression was applied for variables with more than 2 categories. A *P* value less than .05 was considered statistically significant. Statistical analyses were performed using R++ software (Toulouse, France, https://www.rplusplus.com/en/home).

## Results

### Study selection

**Figure 1:**
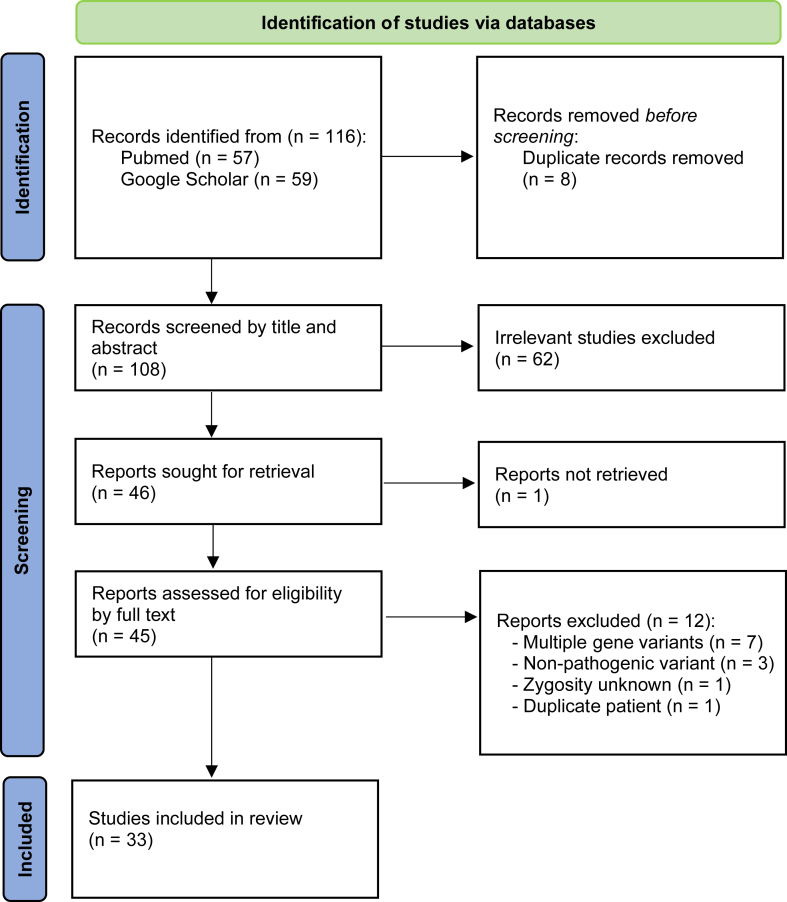
Flowchart of literature search and screening.

A comprehensive literature search identified a total of 116 articles from PubMed (n = 57) and Google Scholar (n = 59) (Figure 1). After excluding 8 duplicates, 108 unique records were screened on the basis of titles and abstracts. During this screening, 1 article was not retrieved and 62 other records were excluded for not meeting the inclusion criteria. Consequently, 45 studies were reviewed at the full-text level, with 12 excluded (7 for including multiple gene variants, 3 for nonpathogenic variants, 1 with unknown zygosity, and 1 with a patient already included) (Table 1).^6^^,^^9^^,^^10^^,^^15^, ^16^, ^17^, ^18^, ^19^, ^20^, ^21^, ^22^, ^23^, ^24^, ^25^, ^26^, ^27^, ^28^, ^29^, ^30^, ^31^, ^32^, ^33^, ^34^, ^35^, ^36^, ^37^, ^38^, ^39^, ^40^, ^41^, ^42^, ^43^, ^44^

**Table 1:** Characteristics of the included studies

Study	Age at first symptom, P	First symptom	Stroke	CA/HF	Atrial standstill	ECG	Conduction abnormalities	TTE	Device	Variant
Alkorashy and colleagues^15^	4 Patients: 19-y-o girl 17-y-o boy 1-y-o boy, P 16-y-o boy	NA NA Syncope NA	No No No Yes	No/Yes No/No No/No No/No	Yes No No No	SND, LQT interval 535 ms SND, LQT interval 558 ms SND, QTc interval 449 ms SND, LQT interval 457 ms	Complete heart block No No No	Severe TR, DA EF 50% EF 50%, DA Normal	PM PM PM PM	***Hom*** p.Cys1850Arg in all 4
Tan and colleagues^16^	2 Patients: 9-y-o girl, P 4-y-o girl	Syncope Asympt	No No	No/No No/No	Yes Yes	SND SND	No No	Normal NA	PM No	***Hom*** p.Val1340Leu
Lopez and colleagues^17^	2 Patients: 6-y-o girl, P 6-y-o girl (twin)	NA Syncope	No No	Yes (PVT and VF)/No No/No	Yes Yes	SND, AT SND	IV conduction delay No	EF 20% after CA	ICD ICD	***Hom*** c.1924A>T
Neu and colleagues^18^	4 Patients: 6-y-o boy, P 4-y-o girl Girl Girl	Dyspnea NA NA NA	No No No No	Yes/Yes No/No No/No No/No	No No No No	SND, VT SND, VT SND, VT SND	RBBB, prolonged PR interval RBBB, prolonged PR interval RBBB, prolonged PR interval RBBB, prolonged PR interval	Ebstein, NC, ↓EF Normal Normal Normal	PM PM PM No	***Hom*** p.Ile230Thr in all
Frigo and colleagues^19^	25-y-o boy, P	NA	No	No/No	No	Brugada syndrome, VT	RBBB, prolonged PR interval	Dilated RV	No∗	***Hom*** p.Arg814Gln
Wang and colleagues^20^	2 Patients: 5-mo-o boy, P NA	Lethargy Asympt	No No	No/No No/No	NA NA	Brugada syndrome, VT, AT SND	Prolonged PR interval Prolonged PR interval and QRS duration	NA NA	ICD No	***Hom*** p.Arg1309His
De Regibus and colleagues^21^	18-y-o girl, P	Palpitations	Yes	No/No	Yes	SND, AFL and AF	No	Biatrial dilation	PM	***Hom*** p.Arg219Cys
Juang and colleagues^22^	3 Patients	NA	No	NA/NA	NA	Brugada syndrome	NA	NA	NA	***Hom*** p.Arg551Thr, p.Asn592Lys p.Arg1193Gln
Lupoglazoff and colleagues^23^	5-y-o boy, P	Syncope	No	No/No	No	LQT interval 526 ms	2:1 Functional AVB	Normal	No	***Hom*** p.Val1777Met
Baskar and colleagues^24^	11-y-o girl, P	Syncope	No	No/No	Yes	SND	Prolonged QRS duration	NA	PM	***Com.Het*** p.Pro1048SerfsX97 and p.Thr220Ile
Benson and colleagues^6^	5 Patients: 6-y-o boy, P 7-y-o boy 2-y-o girl 9-y-o boy, P 5-y-o boy, P	NA NA NA Syncope NA	No No No No No	No/No No/No No/No No/No No/No	Yes Yes NA Yes Yes	SND SND SND SND, VT SND	Prolonged QRS duration Prolonged QRS duration NA Prolonged QRS duration Prolonged QRS duration	Normal Normal Normal Normal Normal	PM PM PM ICD PM	***Com.Het*** p.Gly1408Arg and p.Pro1298Leu in the first 3 ***Com.Het*** p.Thr220Ile and p.Arg1623X ***Com.Het*** del p.Phe1617 and p.Arg1632His
Bezzina and colleagues^9^	2 Patients: Newborn boy, P 1-y-o girl	Severe distress CA	No No	No/No Yes/No	NA NA	Wide QRS tachycardia Wide QRS tachycardia	Broad P waves, prolonged PR interval and QRS duration Broad P waves, prolonged PR interval and QRS duration, 2:1 AVB	Small VSD NA	No No	***Com.Het*** p.Trp156X and p.Arg225Trp in both
Kodama and colleagues^25^	5-y-o boy, P	Asympt	No	No/No	Yes	SND, AF	Prolonged PR interval	Normal	PM	***Com.Het*** p.Asp349Asn and p.Asp1790Asn
Medeiros-Domingo and colleagues^26^	2-y-o boy, P	Syncope	No	Yes/No	NA	LQT interval 519 ms, VT	No	Normal	No	***Com.Het*** p.Arg34fs/60 and p.Arg1195His
Sacilotto and colleagues^27^	4-y-o boy, P	Syncope	No	No/No	No	SND, Brugada syndrome, AF, wide QRS tachycardia	Prolonged PR interval, increased HV interval	Normal	PM	***Com.Het*** p.Gly400Arg and p.Thr1461Ser
Selly and colleagues^28^	10-y-o boy, P	Asympt	No	No/No	NA	SND, AFL	HV interval 72 ms	Normal	PM	***Com.Het*** p.Ala735Val and p.Asp1792Asn
Thongnak and colleagues^29^	18-mo-o girl, P	NA	No	NA/No	NA	Complete AVB	Complete AVB	NA	PM	***Com.Het*** p.Arg34His and p.Val1278Ile
Tan and colleagues^30^	11-y-o boy, P	Syncope	No	No/No	No	Brugada syndrome, polymorphic VT	Prolonged PR interval and QRS duration	Normal	No	***Com.Het*** p.Ala226Val and p.Arg1629X
Cordeiro and colleagues^31^	Boy, P	Asympt	No	No/No	NA	Brugada syndrome	Prolonged PR interval	NA	ICD	***Com.Het*** p.Pro336Leu and p.Ile1660Val
Robyns and colleagues^32^	2 Patients: 14-y-o girl, P 13-y-o boy	Palpitation NA	No No	No/No No/No	NA Yes	SND, AFL SND, AFL	Prolonged HV interval Prolonged QRS duration	Normal NA	PM PM	***Com.Het*** p.Arg1632His and p.Met858Leu in both
Howard and colleagues^33^†	3 Patients: 6-y-o boy, P 4-y-o girl, P 10-y-o boy, P	NA NA NA	Yes No No	NA/NA NA/NA NA/NA	Yes Yes Yes	Brugada syndrome, AFL, VT, VF JT AT, VT	NA NA NA	NA NA NA	ICD PM ICD	***Hom*** p.Arg1309His ***Com.Het*** p.Leu227Pro and p.Met1793Lys ***Com.Het*** p.Ala735Val and p.Arg1512Trp
Abe and colleagues^34^	4-y-o boy, P	Syncope	No	No/No	Yes	SND	No	NA	PM	***Com.Het*** c.2401_2409delinsTCC and P.Met1880Val
Calloe and colleagues^35^	44-y-o man, P	Syncope	Yes	Yes/No	NA	Brugada syndrome, VT	Prolonged PR interval	NA	ICD	***Com.Het*** p.Arg620His and p.Arg811His
Moreau and colleagues^36^	9-y-o boy, P	Pre syncope	No	No/No	Yes	SND, AFL and AF	No	Normal	PM	***Com.Het*** c.1141-2 A>G and p.Pro1053Lys
Villarreal-Molina and colleagues^37^	3 Patients: 22-mo-o boy P 4-y-o boy, P 3-y-o girl, P	CA Syncope Syncope	No No No	Yes/No Yes/No Yes/No	NA NA NA	LQT interval 519 ms, VT SND, VT, AF, LQT interval 495 ms SND, VT, Brugada syndrome, AF and AFL, LQT interval 630 ms	Yes RBBB, prolonged PR interval RBBB and LBBB, third-degree AVB,	NA NA NA	No PM PM	***Com.Het*** pArg34fs∗60 and pArg1195His p.Asp1741Glyfs∗48 and pVal240Met p.Trp1345_Ser1349delinsPhe and p.Pro1730Leu
Sommariva and colleagues^38^	3-y-o boy, P	Asympt	No	No/No	No	Brugada syndrome, VT	prolonged PR interval	Normal	No	***Com.Het*** c.3258_3261del4 and p.Phe1293Ser
Nijak and colleagues^39^	2-y-o boy, P	Syncope	Yes	No/No	Yes	SND, Brugada syndrome	No	Normal	ICD	***Com.Het*** c.4813+3_4813+6dupGGGT and p.Phe1571Leu
Nof and colleagues^40^	16-y-o boy, P	Syncope	No	No/No	No	SND	HV interval 75 ms	Normal	PM	***Com.Het*** p.Lys1493del and p.Ala1924Thr
Wang and colleagues^41^	2 Sisters: 5-y-o, P 12-y-o	REEC NA	No No	No/No NA/No	NA NA	SND SND	No NA	Normal NA	PM PM	***Com.Het*** p.Arg965Cys and p.Arg811Cys in both
Lin and colleagues^42^	2 Patients: 10-y-o boy, P 41-y-o man	Syncope NA	No Yes	No/No No/No	No Yes	SND, AF SND, AF	Third-degree AVB Third-degree AVB	NA NA	PM PM	***Com.Het*** p.Arg965Cys and p.Arg1309His in both
Baruteau and colleagues^10^	3 Patients: <1-y-o, P <1-y-o, P >1-y-o, P	NA NA NA	NA NA NA	NA/NA NA/NA NA/NA	NA NA NA	NA NA NA	NA NA NA	NA NA NA	NA NA NA	***Com.Het*** exact variant NA
Chockalingam and colleagues^43^	2 Families: A male infant P and his sibling A male neonate P and his sister	NA NA NA NA	NA NA NA NA	Yes/No No/No Yes/No Yes/No	NA NA NA NA	NA NA NA NA	NA NA NA NA	NA NA NA NA	NA NA NA NA	***Com.Het*** exact variant NA
Postema and colleagues^44^	46-y-o man, P	NA	No	No/No	NA	LQT interval 500 ms, Brugada syndrome	Prolonged PR interval and QRS duration	NA	No	***Com.Het*** Ile1660Val and ΔKPQ variant

AF = atrial fibrillation; AFL = atrial flutter; Asympt = asymptomatic; AT = atrial tachycardia; AVB = atrioventricular block; CA = cardiac arrest; Com.Het = compound heterozygous; DA = dilated atria; ECG = electrocardiography; EF = ejection fraction; HF = heart failure; Hom = homozygous; ICD = intracardiac defibrillator; IV = intraventricular; JT = junctional tachycardia; LBBB = left bundle branch block; LQT = long QT; mo-o = month-old; NA = not available; NC = noncompaction; P = proband; PM = pacemaker; PVT = polymorphic ventricular tachycardia; QTc = corrected QT; RBBB = right bundle branch block; REEC = reduced endurance exercise capacity; RV = right ventricle; SND = sinus node dysfunction; TR = tricuspid regurgitation; TTE = transthoracic echocardiography; VF = ventricular fibrillation; VSD = ventricular septal defect; VT = ventricular tachycardia; y-o = year-old.

∗ Patient refused ICD implantation.

† Patients from this cohort already reported in other cited articles have been excluded.

### Baseline characteristics

We selected 33 articles for inclusion in our systematic review, comprising a total of 61 patients (35 males [65%]; median age 9 ± 10 years) from 43 distinct families. Most patients, especially those 10 years or younger (*P* = .04) (Table 2), were probands (40/58, 69%) and symptomatic (29/36, 81%) at diagnosis. The most common first clinical presentations were syncope (15/36, 42%) and CA (10/36, 28%). SND was described in the majority (35/54, 65%) of patients, including 20 with atrial standstill (59%). SND was rarely isolated (14/52, 27%) but usually associated with other ECG phenotypes. The 4 other ECG phenotypes were PCCD (17/52, 33%), BrS (12/52, 23%), overlap phenotype (7/52, 13%), and LQTS (2/52, 4%). Patients with isolated SND were predominantly male (*P* = .02) (Table 3).

**Table 2:** Clinical characteristics according to age at diagnosis

Characteristic	Age ≤10 y (n = 36)	Age >10 y (n = 14)	*P*
Clinical characteristics			
Sex (47): male	22 (67)	9 (64)	>.99
Proband (50)	30 (83)	7 (50)	.04
Mode of presentation at diagnosis (31)			.3
Asymptomatic	5 (21)	0 (0)	
Syncope	12 (50)	3 (43)	
CA	5 (21)	2 (29)	
Others	2 (8)	2 (29)	
ECG phenotypes (47)			
LQT3	2 (6)	0 (0)	.9
BrS	5 (15)	3 (21)	.9
PCCD	14 (42)	4 (29)	.6
Isolated SND	9 (27)	5 (36)	.8
Overlap phenotype	3 (9)	4 (29)	.2
Atrial standstill (32)	15 (68)	5 (50)	.6
Arrhythmias (47)			
SVT	9 (27)	4 (29)	>.99
VT	14 (42)	3 (21)	.3
Device (47)	26 (79)	11 (79)	>.99
Heart failure (44)	1 (3)	1 (7)	>.99
MCE (40)	16 (57)	4 (33)	0.3
Stroke (47)	2 (6)	4 (29)	0.1
Death (47)	4 (12)	1 (7)	>.99
Zygosity (50)			.8
Homozygous	10 (28)	26 (72)	
Compound heterozygous	5 (36)	9 (64)	
Variant type (47)			.6
Biallelic missense pathogenic variant	22 (67)	11 (33)	
Missense + non-missense pathogenic variant	11 (79)	3 (21)	

Values are presented as n (%).

BrS = Brugada syndrome; CA = cardiac arrest; ECG = electrocardiographic; LQT3 = long QT syndrome type 3; MCE = major cardiac event; PCCD = progressive cardiac conduction disease; SND = sick sinus node dysfunction; SVT = supraventricular tachycardia; VT = ventricular tachycardia.

**Table 3:** Clinical characteristics according to ECG phenotypes

Characteristic	LQT3	BrS	PCCD	Isolated SND	Overlap phenotype	*P*
Sex (48): male	0 (0)	9 (100)	11 (69)	4 (29)	5 (71)	.02
Proband (49)	2 (100)	9 (100)	10 (59)	9 (64)	3 (43)	.04
Syncope (31)	2 (100)	5 (71)	3 (33)	6 (60)	2 (67)	.5
CA (46)	1 (50)	1 (13)	2 (13)	1 (8)	3 (43)	.6
Arrhythmias						
SVT (52)	0 (0)	3 (25)	3 (18)	5 (36)	2 (29)	.3
VT (52)	1 (50)	7 (58)	6 (35)	0 (0)	3 (43)	.1
Heart failure (48)	0 (0)	0 (0)	1 (6)	0 (0)	1 (14)	.4
MCE (42)	2 (100)	5 (71)	4 (31)	6 (46)	3 (43)	.2
Stroke (52)	0 (0)	3 (25)	1 (6)	1 (7)	1 (14)	.5
Death (49)	1 (50)	0 (0)	2 (12)	0 (0)	2 (29)	.9

Values are presented as n (%).

BrS = Brugada syndrome; CA = cardiac arrest; ECG = electrocardiographic; LQT3 = long QT syndrome type 3; MCE = major cardiac event; PCCD = progressive cardiac conduction defect; SND = sinus node dysfunction; SVT = supraventricular tachycardia; VT = ventricular tachycardia.

### Clinical outcomes

Follow-up periods varied, with a median of 5 ± 7 years. Arrhythmias were frequent, with atrial arrhythmias in 13 (24%) and ventricular arrhythmias in 18 (33%) patients. Nine patients received antiarrhythmic treatment, and 3 received anticoagulation. A device was implanted in 76% of patients (pacemaker: n = 30; implantable cardioverter-defibrillator: n = 9) at a mean age of 12 ± 11 years. Eight patients (15%) died at a mean age of 9 ± 15 years. Of note, 2 patients who initially received a pacemaker experienced CA later in the course: one developed ventricular tachycardia followed by heart failure and ventricular fibrillation,^18^ and the other presented with ventricular tachycardia and ventricular fibrillation during hospitalization.^37^ Other complications such as heart failure (4%) and stroke (11%) were also frequent in this young population. Among the reviewed cases, stroke occurred in 11% of patients. Those who experienced stroke appeared to be diagnosed later (21 ± 18 years vs 8 ± 8 years; *P* = .06). There was no significant correlation between stroke and ECG phenotype, zygosity (*P* = .7), or variant type (*P* > .99). The presence of atrial standstill or supraventricular tachycardia did not significantly influence stroke occurrence (55% vs 80%; *P* = .4 and 21% vs 50%; *P* = .1, respectively), likely owing to the small number of patients and the short follow-up duration (atrial standstill: 10 ± 9 years with 8 ± 8 years of follow-up; supraventricular tachycardia: 11 ± 11 years with 7 ± 6 years of follow-up). Notably, 3 of the 6 patients who suffered a stroke had both supraventricular tachycardia and atrial standstill.

### Genotype-prognosis correlation

Of the 61 patients, 21 (34%) had homozygous variants and 40 (66%) compound heterozygous variants. Consanguinity was identified in a majority (57%) of patients with homozygous *SCN5A* variants. Overall, variants were mostly (82 [86%]) located in domains DI–DIV (67 localized to either DI S1–S4, DII S1–S4, DIII S1–S4, or DIV S1–S4 and 15 localized to the S5, P-loop, and S6 regions containing the pore filter of the sodium channel). Three variants (3%) were localized in N-terminal domains and 10 (11%) in C-terminal domains. Zygosity was not a prognostic factor, in contrast to variant type (Tables 4 and 5). Most variants were missense (40/54, 74%), whereas 26% (14/54) were not (truncation variants: 10%; in-frame variants: 4%). A non-missense variant was more frequently identified in probands (*P* = .04) and younger patients (*P* = .01) (Table 5). The risk of ventricular tachycardia and MCEs was significantly increased in patients with a non-missense variant (*P* = .008 and *P* = .01, respectively).

**Table 4:** Risk of MCEs

Characteristic	No MCE (n = 23)	MCE (n = 23)	*P*
Clinical characteristics			
Sex (45): male	15 (68)	17 (74)	.9
Proband (46)	11 (48)	21 (91)	.003
Age at diagnosis (y)	12 ± 12	8 ± 10	.2
Age ≤ 1 y at diagnosis (44)	1 (5)	4 (17)	.3
ECG phenotypes (42)			
LQT3	0 (0)	2 (10)	.4
BrS	2 (9)	5 (25)	.3
PCCD	10 (45)	6 (30)	.5
Isolated SND	7 (32)	6 (30)	>.99
Overlap phenotype	4 (18)	3 (15)	>.99
Atrial standstill (27)	10 (71)	7 (54)	.4
SVT (42)	5 (23)	7 (35)	.6
Zygosity (46)			.5
Homozygous	8 (35)	5 (22)	
Compound heterozygous	15 (65)	18 (78)	
Variant type (42)			.01
Biallelic missense pathogenic variant	19 (86)	9 (45)	
Missense + non-missense pathogenic variant	3 (14)	11 (55)	

Values are presented as mean ± standard deviation or n (%).

BrS = Brugada syndrome; ECG = electrocardiographic; LQT3 = long QT syndrome type 3; MCE = major cardiac event; PCCD = progressive cardiac conduction disease; SND = sinus node dysfunction; SVT = supraventricular tachycardia.

**Table 5:** Clinical characteristics according to *SCN5A* variant zygosity and variant type

Characteristic	Homozygous (n = 21)	Compound heterozygous (n = 40)	*P*	Missense (n = 40)	Missense + non-missense (n = 14)	*P*
Clinical characteristics						
Sex (54, 50): male	8 (47)	27 (73)	.1	21 (58)	11 (79)	.3
Proband (58, 51)	9 (50)	31 (78)	.07	22 (59)	13 (93)	.04
Age at diagnosis (y)	9 ± 7	9 ± 12	.8	12 ± 12	5 ± 5	.01
Age ≤1 y at diagnosis (54, 47)	2 (13)	5 (13)	1	2 (6)	2 (14)	.6
Mode of presentation at diagnosis (36, 33)			.4			.3
Asymptomatic	3 (27)	4 (16)		6 (30)	1 (8)	
Syncope	5 (45)	10 (40)		8 (40)	7 (54)	
CA	1 (9)	9 (36)		3 (15)	4 (31)	
Others	2 (19)	2 (8)		3 (15)	1 (8)	
ECG phenotypes (54, 54)						
LQT3	1 (5)	1 (3)	1	1 (3)	1 (7)	>.99
BrS	6 (29)	6 (18)	.6	9 (23)	3 (21)	>.99
PCCD	8 (38)	13 (39)	1	15 (38)	6 (43)	>.99
Isolated SND	6 (29)	8 (24)	1	12 (30)	2 (14)	.4
Overlap phenotype	3 (14)	4 (12)	1	4 (10)	3 (21)	.5
Atrial standstill (34, 34)	7 (44)	13 (72)	.2	15 (58)	5 (63)	>.99
Arrhythmias (54, 54)						
SVT	4 (19)	9 (27)	.7	11 (28)	2 (14)	.5
VT	6 (29)	12 (36)	.8	9 (23)	9 (64)	.008
Device (51, 51)	13 (72)	26 (79)	.9	31 (84)	8 (57)	.07
Heart failure (52, 48)	2 (12)	0 (0)	.2	2 (6)	0 (0)	.9
MCE (46, 42)	5 (38)	18 (55)	.5	9 (32)	11 (79)	.01
Stroke (54, 54)	3 (14)	3 (9)		5 (13)	1 (7)	>.99
Death (55, 51)	2 (11)	8 (16)	.9	2 (5)	3 (21)	.2

Values are presented as mean ± standard deviation or n (%).

BrS = Brugada syndrome; CA = cardiac arrest; ECG = electrocardiographic; LQT3 = long QT syndrome type 3; MCE = major cardiac event; PCCD = progressive cardiac conduction disease; SND = sinus node dysfunction; SVT = supraventricular tachycardia; VT = ventricular tachycardia.

## Discussion

SND and PCCD were the most prevalent phenotypes. Looking more broadly across all 61 cases, we showed that the phenotype was severe, with MCEs in half of the cases, especially in patients with non-missense *SCN5A* variants.

### Cardiac phenotype of patients with biallelic *SCN5A* variants

Having homozygous or compound heterozygous *SCN5A* variants is a rare condition, representing less than 2% of all patients with *SCN5A* variants across different cohorts.^10^^,^^45^ A high degree of phenotypic variability has been associated with heterozygous *SCN5A* variants owing to their effect on Na_V_1.5 biophysical properties^46^ and other modulating factors such as age, sex, or time of day. Gain-of-function variants are associated with LQTS type 3,^2^ whereas loss-of-function variants are associated with BrS^4^ or abnormal cardiac conduction. This pleiotropy of cardiac phenotypes was also observed in patients with biallelic *SCN5A* variants, with BrS in 12 (23%), LQTS in 2 (4%), and overlap phenotype in 7 (13%) patients. Conduction defects were frequently observed, with atrial standstill in 20 (59%), SND in 35 (65%), and PCCD in 17 (33%) patients. Although our findings support a key role for *SCN5A* variant type in determining phenotype severity, they also fit within a broader and evolving understanding of BrS as a complex disorder not solely explained by rare monogenic variants. Recent genome-wide association studies^47^ have shown that common genetic variants, quantified using polygenic risk scores, may contribute to susceptibility to the Brugada ECG pattern, particularly in the absence of a rare *SCN5A* variant. This suggests that *SCN5A*-independent mechanisms may play a role in disease expression and supports a more integrated approach to genetic risk assessment. Interestingly, beyond genetic variants, recent evidence^48^ suggests that immunological mechanisms may modulate sodium channel function. In particular, the presence of anti-Na_V_1.5 autoantibodies has been reported in the majority of patients with BrS, including those without *SCN5A* variants. These autoantibodies were shown to reduce sodium current density and reproduce Brugada-like ECG abnormalities in animal models, suggesting an immune-mediated contribution to disease expression. This emerging concept may help explain the phenotypic variability observed even among individuals sharing the same *SCN5A* genotype and represents a potentially confounding factor when interpreting genotype-phenotype correlations.

### Therapeutic management of patients with biallelic *SCN5A* variants

Three types of complications can occur in patients with biallelic *SCN5A* variants: cardioembolic, arrhythmic (SND with bradycardia or ventricular arrhythmias), and hemodynamic complications. CA occurred in 28%, and a device was implanted in three-quarters of cases. The indication for pacemaker or implantable cardioverter-defibrillator implantation followed standard guidelines, with a young mean age at implantation (12 ± 11 years). Of note, 2 patients who initially received a pacemaker experienced CA later in the course: one developed ventricular tachycardia followed by heart failure and ventricular fibrillation,^18^ and the other presented with ventricular tachycardia and ventricular fibrillation during hospitalization.^37^ This observation suggests that pacemaker implantation may not provide sufficient protection against ventricular arrhythmias in some cases, reinforcing the importance of careful risk stratification. These results corroborated previous studies demonstrating that patients with biallelic *SCN5A* variants present with a more severe phenotype, experience more cardiac events, and are diagnosed at a younger age at diagnosis.

Furthermore, our results demonstrated a high occurrence of stroke (11%). Moreau and colleagues^36^ recently highlighted the association between *SCN5A* variants and stroke in young patients. This association was described in 2 children aged 11 and 14 years, both with severe atrial arrhythmias. The 14-year-old patient also exhibited atrial standstill. In our study, only 3 patients received anticoagulation, and 59% and 24% had atrial standstill and atrial arrhythmias, respectively. These findings raise the question of whether anticoagulation should be considered in these specific patients.

### Genotype-phenotype correlation

Zygosity and variant type were not significantly correlated with ECG phenotype. However, unlike zygosity, variant type affected the risk of MCEs. Indeed, 79% of patients with a non-missense variant experienced MCEs (vs 32% with missense variants; *P* = .01). Also, non-missense variants were more frequently identified in probands (*P* = .04) and younger patients (*P* = .01). Importantly, no patients with 2 non-missense variants have been reported in the literature, suggesting that this genotype may be incompatible with life. This hypothesis is supported by experimental studies^49^ and underlines the severity of phenotypes associated with a single non-missense variant. This is an important finding that may have significant implications for refining therapeutic strategies in affected patients and their relatives.

### Limitations

This study had several limitations and potential biases. First, the primary limitation is that data analysis was based on incomplete information. Not all articles consistently reported clinical data, such as sex, first symptoms, or age at diagnosis. Second, there is publication bias, as an unknown number of patients may have died without undergoing genetic testing. Finally, the patients included in this study had relatively short follow-up periods, limiting insight into long-term disease progression.

## Conclusion

This study provides valuable insights into the clinical spectrum and prognosis of patients with biallelic *SCN5A* variants. These patients are at high risk of life-threatening arrhythmias and stroke from an early age. Given the severity and rarity of this condition, international registries and collaborative research are essential to improve risk stratification, guide personalized management, and ultimately prevent sudden cardiac death in affected individuals.
